# The Masticatory Structure and Function in Children with Cerebral Palsy—A Pilot Study

**DOI:** 10.3390/healthcare11071029

**Published:** 2023-04-04

**Authors:** Karolina Szuflak, Roksana Malak, Brittany Fechner, Dorota Sikorska, Włodzimierz Samborski, Ewa Mojs, Karolina Gerreth

**Affiliations:** 1 Department of Risk Group Dentistry Chair of Pediatric Dentistry, Poznan University of Medical Sciences, 60-812 Poznan, Poland; 2 Doctoral School, Poznan University of Medical Sciences, 60-812 Poznan, Poland; 3 Department and Clinic of Rheumatology, Rehabilitation, and Internal Medicine, Poznan University of Medical Sciences, 61-545 Poznan, Poland; 4 Department of Clinical Psychology, Poznan University of Medical Sciences, 60-812 Poznan, Poland

**Keywords:** masticatory muscles, strain-counterstrain technique (SCS), NeuroDevelopmental Treatment-Bobath (NDT-Bobath) method, salivation, cerebral palsy (CP)

## Abstract

(1) Background: Muscle tension around the head and neck influences orofacial functions. The data exist concerning head posture during increased salivation; however, little is known about muscle tightness during this process. This study aims to investigate whether or not any muscles are related to problems with eating, such as drooling in individuals with cerebral palsy; (2) Methods: Nineteen patients between the ages of 1 and 14 were examined prior to the physiotherapy intervention. This intervention lasted three months and consisted of: relaxing muscles via the strain-counterstrain technique, functional exercises based on the NeuroDevelopmental Treatment-Bobath method, and functional exercises for eating; (3) Results: the tone of rectus capitis posterior minor muscle on the left side (*p* = 0.027) and temporalis muscle on the right side (*p* = 0.048) before the therapy, and scalene muscle on the right side after the therapy (*p* = 0.024) were correlated with drooling behavior and were considered statistically significant. Gross motor function was not considered statistically significant with the occurrence of drooling behavior (*p* ≤ 0.05). Following the therapeutic intervention, the frequency of drooling during feeding decreased from 63.16% to 38.89% of the total sample of examined patients; (4) Conclusions: The tightness of the muscles in the head area can cause drooling during feeding.

## 1. Introduction

Drooling (sialorrhea) is defined as an involuntary flow of saliva from the mouth. After the age of 18 months, it is very often associated with intellectual disability and neuromuscular disorders such as cerebral palsy (CP) [1,2,3,4,5]. Approximately 90% of patients with CP also have some form of oral-motor dysfunction [6], of which 10–58% of these individuals have a problem with drooling [5]. This is due to dysfunctional voluntary oral motor activity, improper swallowing, and oral sphincter deficits. Drooling behavior in patients with CP is rarely caused by hypersalivation [7,8,9]. Therefore, causes of oral-motor disorders tend to be variable and challenging in therapeutic contexts. Therapists should conduct very complex examinations and interviews with patients. Also, the observation of feeding behavior should be performed. Only then will patients and families have the benefit of therapy to be thankful for. Rehabilitation and feeding therapy is for patients and their families.

Nowadays, the medical field proposes various treatments, such as botulinum toxin injections, surgical procedures, laser photocoagulation, pharmacotherapy, and acupuncture [2,5,10,11,12,13,14]. It is necessary to include safe complementary therapies, which involve interdisciplinary medical teams. Exploration of this subject may open the way for determining additional therapeutic options. Muscle tone is rarely considered a significant factor that may influence the eating behavior of children. Some researchers recognize the auxiliary muscles used during eating, the tension of which may affect the positioning of the head and the process of feeding [15,16,17]. Nowadays, we still have a shortage of information concerning the tension of muscles around the head or neck and relating to the process of eating and drooling in children.

According to Chávez (2008), Dias (2016) and Gerek (2005), intensified drooling may make it difficult to ingest food [4,5,6]. The lack of such information and the need for help during the feeding process for individuals may cause moderate to severe secondary malnutrition and limit hydration in patients with CP [18,19]. On the other hand, there are some reports about physiotherapy treatment causing decreased drooling in this group of patients. Kumari (2018) proved, when comparing two groups of participants, that oral motor therapy (*p* < 0.0001) was found to be more effective than oral facial facilitation (*p* = 0.0719) [20]. Moreover, Muammer (2010) investigated a combination of physiotherapeutic interventions, such as electrical stimulation and proprioceptive neuromuscular facilitation (PNF), can be helpful in reducing drooling in children with neurodevelopmental disorders [21]. Moreover, Cocks (2022) concluded that if the orbicularis oris is the primary muscle responsible for closing the lips, expiratory muscle strength training (EMST) could contribute to reducing drooling. Therefore, it was decided to prepare an examination to check the influence of expiratory muscle strength training on drooling for people with Parkinson’s disease this showed the significant result that drooling and swallowing improved following training (*p* < 0.01) [22]. Thus, it would be necessary to explore the reason for the occurrence of excessive salivation and drooling in these patients during feeding. This became a research question of whether the muscle tension around the head and neck influences drooling? Therefore, we aimed to study the relationship between excessive drooling during feeding and muscle hypertension and have proposed a complementary treatment for sialorrhea.

## 2. Materials and Methods

We obtained permission from the Bioethics Commission at the Poznan University of Medical Sciences (ref. No. 339/15, dated 9 April 2015). Our study group included children with CP. The type in all subjects’ neurological classification of cerebral palsy was quadriplegic. These patients were individuals living in a social welfare home, so their medical records were largely unknown. Even a medical history was also very often unclear. We included only this aspect which was retrospective and capable of examination. The intervention took place in 19 patients (11 males and 8 females; aged 1–14, mean age ± SD = 6.21 ± 2.82) during the first examination and before the start of the therapeutic intervention. A total of 18 children were assessed during the second examination following the end of the treatment. One participant was a 14-year-old female who was hospitalized during the second examination, and therefore could not be evaluated. The entire examined group presented the level V in the Gross Motor Function Classification System (GMFCS). GMFCS, as a valid 5- level system and reliable in 85% of cases, serves to classify the severity of motor function in pediatric patients with disability [23]. This means that the patient was not able to exhibit head and trunk control, nor any locomotion activities [8,9,24]. The inclusion criteria were difficulties with sucking, swallowing, and excessive salivation during eating according to the Castillo Morales Questionnaire [3,25]. We only used part of the Castillo Morales Questionnaire as it was not fully translated into the Polish language. Similarly we assessed whether children presented excessive drooling according to The Drooling Quotient Assessment (DQ) of which the reliability is 95% [26]. Patients presented the V level in the Eating and Drinking Ability Classification System (EDACS) which mean that the patient is unable to eat and drink safely—tube feeding may be considered to provide nutrition [27]. We excluded patients with inflammatory processes, tumors, any lethal diseases, and any patients who received any prior interventions to reduce drooling. Please see Table 1 below for additional inclusion and exclusion criteria (Table 1).

We developed the therapeutic intervention based on the strain-counterstrain technique (SCS) and NeuroDevelopmental Treatment-Bobath (NDT-Bobath). SCS is an osteopathic manipulative technique of soft tissues successfully used to relieve musculoskeletal pain and improve dysfunction mobility restrictions [27,28]. NDT-Bobath treatment was developed for treating neurodevelopmental disorders in young and adult people. Since 1940, NDT-Bobath has been based on research about brain functions and neurophysiology [29]. This treatment also included posture and balance training, which aims to improve children to the maximum independence level in each activity daily of living [29].

### 2.1. The Examination

The examination was prepared and carried out by experienced and trained physiotherapists. During the first examination before the therapeutic intervention and also during the second examination after the therapeutic intervention, physiotherapists assessed the muscle tone of each patient’s masticatory muscles and auxiliary muscles. The assessor compared muscle tension between the right and left sides of the body by applying a gentle touch when palpating the muscle at a tender point. Each participant was diagnosed using the Gross Motor Function Measure–88 (GMFM-88). This measurement is the gold standard for measuring the quantitative evaluation of motor function in children with cerebral palsy from 5 months to 15 years old. This evaluative tool measures the effectiveness of therapy in improving gross motor function [30,31,32]. Using the same score of GMFM-88 in a study, we can compare the results of participants from different age groups. This is the reason why we included patients between 1 to 14 years old. At the beginning and end of the study, the examiners also assessed participants with The Drooling Quotient Assessment if drooling appeared and made the feeding of participants difficult.

### 2.2. The Therapeutic Intervention

Subsequently, we performed our therapeutic intervention, which lasted three months. During each day of therapy (two days per week), physiotherapists palpated the muscle tone of the following muscles: masseter, temporalis, sternocleidomastoid, trapezius, scalene, serratus anterior, and rectus capitis posterior minor. For the examination procedure, each patient was placed in a supine position, with the patient’s back situated in a comfortable position to ensure the relaxation of the muscles. The therapy involved relaxing muscles responsible for sucking, swallowing, and chewing via the strain-counterstrain (SCS) technique and using the NeuroDevelopmental Treatment-Bobath (NDT-Bobath) method, which supported the proper positioning of the child while feeding the participant and the facilitation of functional sucking and swallowing, and chewing. Then we performed the SCS to relax the tensed muscles. For example, the assessment and therapy of the masseter muscle is carried out with the child’s mouth open with a slight translation of the jaw towards the muscle, or the treatment of the sternocleidomastoid muscle takes place with the lateral flexion of the head to one side and rotation to the opposite side. When a physiotherapist selected the most tightened muscle, he put his index finger gently positioned with constant pressure for 90 s based on the SCS [33]. Two of the tensest muscles were relaxed during each treatment. After the SCS was applied, we supported each patient in a proper sitting position and facilitated eating based on the NDT-Bobath. After the period of three months with the therapeutic interventions took place, the second examination assessed the tension of the muscles and gross motor function by GMFM-88 again, and drooling as part of the Drooling Quotient Assessment.

Data were analyzed using STATISTICA 8.1 (StatSoft). To analyze the relationship between muscle tone and the presence of a given function, we used Pearson’s chi-squared test. The Mann-Whitney test was used to determine the relationship between the test results according to the GMFM-88 and the occurrence of drooling. Correlation between samples was measured using Spearman’s rank correlation coefficient. The results were considered statistically significant at *p* ≤ 0.05.

## 3. Results

### 3.1. Muscle Tone and Drooling

There was a statistically significant correlation between the occurrence of excessive salivation before the therapy and the hypertonicity of the rectus capitis posterior minor muscle on the left side (*p* = 0.027) and temporalis muscle on the right side (*p* = 0.048) (Table 2). We did not observe this correlation until after the therapy. However, the correlation after the therapy between tightness and the occurrence of excessive salivation was considered statistically significant in the scalene muscle on the right side (*p* = 0.024). It is worth noting that about 50% of patients with proper scalene muscle tension did not have drooling. Overall, we observed the tendency of tightness of the scalene muscle and excessive salivation (Table 3). The tension of the remaining muscles was not significantly correlated with drooling.

### 3.2. Motor Function and Drooling

The results from the GMFM-88 were not statistically significantly or correlated with drooling (before therapy *p* = 0.375, after therapy *p* = 0.928). After treatment, the frequency of drooling during feeding decreased from 63.16% to 38.89% of patients (Table 4).

## 4. Discussion

We observed tightness in muscles that are related to sialorrhea. Our results show that our suggested therapy treatment positively affected food intake behavior.

We consider the SCS technique to be a useful and quick tool for facilitating muscle relaxation in children. It is painless, and as a result younger patients tended to accept it very well. Furthermore, the shorter time of stimulation ensured that patients are neither so weary nor restricted in their free movement [34]. We can palpate the tensed muscle and, using the SCS, relax it to improve body posture or treat the dysfunction. The NDT-Bobath method is a good choice of treatment since this method seeks to improve gross motor function, especially in children with CP. We also chose this method since it takes into consideration balance and postural control [29]. As previously mentioned, postural control is essential during feeding procedures. According to Acar (2021), the NDT-Bobath method has exercises that may supplement feeding therapy that may improve swallowing and oral motor skills in children with CP [35]. These results should be taken into account when considering how to comprehensively conduct therapeutic interventions with patients who have feeding problems. Our recommended therapy was effective in decreasing drooling. We have shown the following connection between the two therapies: The SCS and NDT-Bobath could be useful in patients with excessive salivation and problems with feeding. Using the above therapies, specialists would ensure their patients’ comprehensive treatment.

During our examination we experienced barriers when feeding our patients with CP, which was also experienced daily by their caregivers. An involuntary loss of saliva made feeding non-effective. After the therapeutic intervention and equalization of muscle tone, the drooling decreased from 63.16% to 38.89% of participants, and the comfort and efficiency of feeding significantly increased for our patients. Gerek (2005) conducted a similar study in which 85.7% (n = 7) of patients experienced poor saliva management prior to treatment [6]. The results from this study indicate significant improvements, among others, in saliva control after the NDT-Bobath method was applied. Another examination aimed to determine if Kinesio Taping (KT) and oral-motor training (OMT) influence drooling in children with intellectual disabilities [36]. The therapy was applied for muscles to orbicularis oris, supra-hyoid, and masseter muscles, and the results showed a significant reduction in drooling post-intervention (*p* < 0.001). There was also a Drooling Quotient Assessment (DQ) used to diagnose the drooling as it appears [36]. Similar therapy, based on strengthening the orbicularis oris and expiratory muscle, was conducted in Parkinson’s disease patients and, likewise, has a positive effect and reduced drooling [22]. These results also suggest the interdependence of muscle tone and the regulation of saliva, which we also observed.

Several authors suggested that the primary influence on oral function has been the following masticatory muscles: masseter, temporalis, medial pterygoids [37,38]. However, we observed a significant correlation between tightness of the temporalis (*p* = 0.048), rectus capitis posterior minor (*p* = 0.027) and scalene muscles (*p* = 0.024) and excessive salivation. Scalene muscles (lac. musculi scaleni) belong to the flexors group of deep neck muscles. Their tension increases when a person has a forward head posture [39]. In this position, the rectus capitis posterior minor muscles (lac. musculus rectus capitis posterior minor) are in the contraction, so they also have increased tension [17]. We found that a forward head posture may increase the occurrence of excessive drooling. As a result, we positioned each patient using the proper chin-tuck position via the NBT-Bobath method. Thanks to this method, we had a continuation of the previous SCS technique on the scalene muscle [40,41,42]. Taş (2015) also found a significant difference between head control and drooling and that the intensity of producing salivation was higher in individuals with poorer head control (*p* = 0.038) [18]. Moreover, the authors proved that when drooling severity increased, the BMI index decreased significantly (*p* = 0.018). In addition, the meaning in the group that had better drooling control, the independence of eating ability, was found to be more significant [18]. Studies presented and our examination show the magnitude of drooling therapy for patients with neurodevelopmental disabilities to improve their quality of life and their health level.

According to Chávez (2008), Lobo (2013), and Linden (1998), muscles that are engaged in head positioning may affect ingestion [4,43,44]. Additionally, the positioning of the head may influence masticatory function. Gadotti (2020) also found this when they checked the activity of masticatory muscles—temporalis and masseter—during chewing in the natural head posture and forward head posture through the use of EMG. Examiners observed a trend of tighter masseter and temporalis muscles during chewing in head protraction. They found a significant increase in masseter muscle activity in the forward head posture [45]. Poor head control appears during lower tension in the postural muscles. Contributing factors to drooling include the sitting position, proper positioning of the head, anatomic and dental malformations, tongue activity, decreased oral sensory awareness, the inability to breathe through the nose, as well as the individual’s emotional state and ability to concentrate [3,5,7]. This is why during our research all patients were properly positioned from the beginning of the study through the facilitation of the NDT-Bobath method. We placed each patient in a seated position with appropriate head posture which helped in the proper placement of the tongue, head, and neck where the respiratory and digestive tracts begin. In patients who are unable to develop head control, another approach is to develop correct sitting and eating postures by training family or caregivers [18]. Therefore, comprehensive therapy should also focus on balance in the postural muscles, especially in those that contribute to head positioning. Examiners from Marmara University in Turkey, observed this significant finding between trunk muscles and oral motor functions in children with CP [35]. According to our intervention, when feeding a child, the following factors should be taken into consideration: drooling, correct sitting position during feeding, and having proper muscle tone. In addition, Haralur (2014) proves in the examination of fifty participants that incorrect head position will result in misalignment of the jaw or even occlusion disorders. The result was a statistically significant difference (*p* < 0.5) between occlusal contacts in three different head postures [46]. We can conclude that the interdisciplinary medical team should treat subjects who have orofacial dysfunctions.

We were also interested in how each patient’s level of GMFCS correlated with drooling. Speyer (2019) concluded in their meta-analysis that a higher GMFCS level was correlated to a higher prevalence of drooling, swallowing, and feeding problems [19]. Conversely, Senner (2004) showed that GMFCS levels were not significantly correlated with the severity of salivation, which also showed in our study [3]. Therefore, we found that a patient’s GMFCS level should not necessarily be associated with excessive salivation. Moreover, our findings suggested that drooling in patients with CP is correlated with swallowing difficulties. Senner (2004) also found that drooling behavior is related to swallowing difficulties rather than to an increased production of saliva in patients with CP [3]. In addition, children suffering from dysphagia may be at risk of aspiration-related lung disease, malnutrition, neurodevelopmental deficits, and social problems, including relationships with caregivers [18,19,47]. We can conclude that drooling appears in patients with neurodevelopmental disorders for numerous reasons, so the treatment should incorporate comprehensive and multidisciplinary teams of medical specialists to improve one of the crucial activities of daily living—eating. This pilot study contributed to the importance of many feeding aspects for therapists and also parents or caregivers of children with neurological problems.

Our study has some limitations. The number of study subjects is small because this is a pilot study, and we are going to expand the group in the future. The group of patients could vary in terms of disability. Other neurodevelopmental disorders could be investigated in a future study. However, our study presents only children with a GMFCS level V because excessive salivation generally appears in these subjects [48]. We could use a more measurable tool to diagnose muscle tension. Other procedures, such as an electromyography (EMG), would be difficult for the patients to tolerate since their functional and medical conditions were characterized as severe disabilities [29]. Furthermore, the electrodes would not stay on the skin due to wet issues of excessive salivation. Therefore, the children did not receive an EMG, which would be a more impartial method for measuring muscle tone.

## 5. Conclusions

Therapeutic interventions for eating should take into consideration muscle tension regulation. Excessive drooling may be related to the tension of some muscles in the neck and head. The therapy we proposed could be helpful as a complementary therapy in medicine in patients experiencing excessive salivation.

## Figures and Tables

**Table 1:** Inclusion and exclusion criteria for participants diagnosed with cerebral palsy.

Inclusion Criteria	Exclusion Criteria
Difficulties with sucking, swallowing, or excessive salivation	No oral motor dysfunctions
Children residing in one selected social welfare home in Poznan	Children residing in other social welfare homes than one selected in Poznan
Children with CP and severe intellectual disability	Children without CP and severe intellectual disability
Written and informed consent from the parent/caregiver for the examination and therapy of the child	No written and informed consent from the parent/caregiver for the examination and therapy of the child
Patient’s cooperation during examination and therapy	Patient uncooperativeness during exam and therapy
Children present at social welfare home on days of examination and therapy	Children absent at social welfare home on days of examination and therapy
Group of children with the same regional, demographic, cultural, and ethnic origin	Group of children with other regional, demographic, cultural, or ethnic origin
Children with a GMFCS level V	Children with a GMFCS level IV or lower
Patients presented the V level in the EDACS	Patients presented the IV level or lower in the EDACS

**Table 2:** The evaluation of dependence between the muscle tension and the occurrence of salivation before therapy (n = 19).

Muscles	Side	Type of Tension	Lack Salivation		Excessive Salivation		*p*
			n	%	n	%	
scalene	left	proper	3	15.79	7	36.84	0.515
		increased	4	21.05	5	26.32	
scalene	right	proper	4	21.05	6	31.58	0.764
		increased	3	15.79	6	31.58	
rectus capitis posterior minor	left	proper	1	5.26	8	42.11	0.027 *
		increased	6	31.58	4	21.05	
rectus capitis posterior minor	right	proper	6	31.58	6	31.58	0.119
		increased	1	5.26	6	31.58	
temporalis	left	proper	5	26.32	5	26.32	0.210
		increased	2	10.53	7	36.84	
temporalis	right	proper	2	10.53	9	47.37	0.048 *
		increased	5	26.32	3	15.79	
masseter	left	proper	5	26.32	5	26.32	0.210
		increased	2	10.53	7	36.84	
masseter	right	proper	3	15.79	6	31.58	0.764
		increased	4	21.05	6	31.58	
sternocleidomastoid	left	proper	4	21.05	6	31.58	0.764
		increased	3	15.79	6	31.58	
sternocleidomastoid	right	proper	2	10.53	6	31.58	0.361
		increased	5	26.32	6	31.58	
trapezius	left	proper	3	15.79	6	31.58	0.764
		increased	4	21.05	6	31.58	
trapezius	right	proper	2	10.53	6	31.58	0.361
		increased	5	26.32	6	31.58	
serratus anterior	left	proper	6	31.58	9	47.37	0.581
		increased	1	5.26	3	15.79	
serratus anterior	right	proper	6	31.58	8	42.11	0.361
		increased	1	5.26	4	21.05	

Pearson’s chi-squared test was used; * statistically significant; the *p*-value was significant at *p* ≤ 0.05; n—number; %—percentage.

**Table 3:** The evaluation of dependence between the muscle tension and the occurrence of salivation after therapy (n = 18).

Muscles	Side	Type of Tension	Lack Salivation		Excessive Salivation		*p*
			n	%	n	%	
scalene	left	proper	8	44.44	4	22.22	0.494
		increased	3	16.67	3	16.67	
scalene	right	proper	9	50.00	2	11.11	0.024 *
		increased	2	11.11	5	27.78	
rectus capitis posterior minor	left	proper	5	27.78	6	33.33	0.088
		increased	6	33.33	1	5.56	
rectus capitis posterior minor	right	proper	9	50.00	3	16.67	0.088
		increased	2	11.11	4	22.22	
temporalis	left	proper	11	61.11	5	27.78	0.060
		increased	0	0	2	11.11	
temporalis	right	proper	4	22.22	4	22.22	0.387
		increased	7	38.89	3	16.67	
masseter	left	proper	8	44.44	6	33.33	0.518
		increased	3	16.67	1	5.56	
masseter	right	proper	7	38.89	3	16.67	0.387
		increased	4	22.22	4	22.22	
sternocleidomastoid	left	proper	6	33.33	3	16.67	0.629
		increased	5	27.78	4	22.22	
sternocleidomastoid	right	proper	8	44.44	4	22.22	0.494
		increased	3	16.67	3	16.67	
trapezius	left	proper	6	33.33	3	16.67	0.629
		increased	5	27.78	4	22.22	
trapezius	right	proper	5	27.78	6	33.33	0.088
		increased	6	33.33	1	5.56	
serratus anterior	left	proper	8	44.44	7	38.89	0.130
		increased	3	16.67	0	0	
serratus anterior	right	proper	9	50.00	6	33.33	0.829
		increased	2	11.11	1	5.56	

Pearson’s chi-squared test was used; * statistically significant; the *p*-value was significant at *p* ≤ 0.05; n—number; %—percentage.

**Table 4:** The comparison of the number of patients presenting salivation before and after therapy.

	Before Therapy		After Therapy	
	n	*%*	n	*%*
patients presenting excessive salivation	12	63.16%	7	38.89%
total patients	19	100%	18	100%
*p*	0.375		0.928	

The Mann-Whitney test was used; the *p*-value was significant at *p* ≤ 0.05; n—number; %—percentage.

## Data Availability

The data that support the findings of this study are available per request from the corresponding author [K.S. and R.M.]. Some data are not publicly available due to containing information that could compromise the privacy of research participants.
